# The Oral Administration of Antitoxin

**Published:** 1907-03

**Authors:** 


### THE ORAL ADMINISTRATION OF ANTITOXIN.

---

The advantages which surround the oral administration of antitoxin in the treatment of diphtheria and tetanus, the two diseases in which antitoxin treatment has so far proved itself advantageous, are so numerous and great as to manifest even to the lay mind, since it cannot be denied that the natural way of introducing substances into the body for effect upon the organism is by the mouth, and that hypodermic injection is possessed of some danger. Further than this, the necessity of preparing antitoxin in such a way that it can be given by the hypodermic syringe adds so greatly to the cost as to prove a serious obstacle to the free use of these valuable therapeutic agents. On the other hand, the few clinicians who have attempted to treat diphtheria and tetanus by the administration of antitoxin by the mouth have rarely obtained sufficiently satisfactory results to make them inclined to repeat the procedure or to recommend it to their less venturesome comrades.

We note with much interest a valuable original research by McClintock and King which is published in the Journal of Infectious Diseases of October 30, 1906, their investigations being made upon animals and men. They find that the administration of antitoxin by the mouth cannot be relied upon to produce results which correspond in value to those which follow its hypodermic use, but they also find that antitoxin is absorbed as such from the stomach, and aids the body materially in combating the infection. A very interesting part of their research is their ability to show that diphtheria and tetanus antitoxin when mixed with trikresol, salol, chloroform and opium are much more effective when they are taken upon an empty stomach, in which the digestive function has been diminished by the simultaneous administration of some drug, than when they are administered to a stomach which is in an active state. In other words, if the digestive juices are present in large quantity they act deleteriously upon the antitoxic substances, and therefore it is important to see, first, that the antitoxin is administered by the mouth during a period of starvation, and second, that several hours must be allowed to elapse for the absorption of these substances before food is taken. The best methods for the purpose of diminishing digestive activity have not as yet been decided by them, but the substances which we have named, and which are commonly mixed with antitoxin for preservative purposes, seem to result in its more speedy absorption than occurs when pure antitoxin alone is given. If any method can be devised by which fairly accurate and reliable results can be obtained from the oral use of these substances, a distinct therapeutic advance will be made, and while the quantity of serum may of necessity be greatly above that now commonly employed by the hypodermic needle, the cost will be markedly diminished because of the simplified method of administration.—Therapeutic Gazette, January, 1907.

